# Evaluation of urinary C-reactive protein as an early detection biomarker for pancreatic ductal adenocarcinoma

**DOI:** 10.3389/fonc.2024.1450326

**Published:** 2024-09-06

**Authors:** Nurshad Ali, Silvana Debernardi, Evelyn Kurotova, Jian Tajbakhsh, Nirdesh K. Gupta, Stephen J. Pandol, Patrick Wilson, Stephen P. Pereira, Bill Greenhalf, Oleg Blyuss, Tatjana Crnogorac-Jurcevic

**Affiliations:** ^1^ Centre for Cancer Biomarkers and Biotherapeutics, Barts Cancer Institute, Queen Mary University of London, London, United Kingdom; ^2^ 3rd Street Diagnostics, Cedars-Sinai, Los Angeles, CA, United States; ^3^ Samuel Oschin Comprehensive Cancer Institute, Cedars-Sinai, Los Angeles, CA, United States; ^4^ Department of Medicine, Cedars-Sinai, Los Angeles, CA, United States; ^5^ Barts Health, Royal London Hospital, London, United Kingdom; ^6^ Institute for Liver and Digestive Health, University College London, London, United Kingdom; ^7^ Department of Molecular and Clinical Cancer Medicine, Institute of Translational Medicine, University of Liverpool, Liverpool, United Kingdom; ^8^ Centre for Cancer Screening, Prevention and Early Detection, Wolfson Institute of Population Health, Queen Mary University of London, London, United Kingdom; ^9^ Department of Pediatrics and Pediatric Infectious Diseases, Institute of Child´s Health, Sechenov First Moscow State Medical University (Sechenov University), Moscow, Russia

**Keywords:** C-reactive protein, LYVE1, TFF1, REG1B, CA19-9, pancreatic cancer, urine

## Abstract

Pancreatic ductal adenocarcinoma (PDAC) is one of the leading causes of cancer-related death worldwide. Up to now, no specific screening or diagnostic tests are available for early PDAC detection. As a result, most patients are diagnosed with advanced or metastatic disease, which leads to a poor prognosis. In this study, we aimed to evaluate the diagnostic value of urinary CRP (uCRP) alone and in combination with our previously established urine biomarker panel (REG1B, LYVE1 and TFF1) for early detection of PDAC. A total of 534 urine samples from multiple centres were analysed: 93 from healthy individuals, 265 from patients with benign hepatobiliary diseases and 176 from PDAC patients. The uCRP and the urinary biomarker panel were assessed using commercial ELISA assays, while plasma CA19-9 and blood CRP (bCRP) were measured using Roche Cobas platform. Multiple logistic regression and nonparametric Kruskal–Wallis test were used for statistical analysis. An internal validation approach was applied, and the validated AUC estimators were reported to ensure accuracy. A significant difference was observed in the medians of uCRP between healthy and benign controls and PDAC sample groups (p < 0.001). uCRP levels were not dependent on gender and age, as well as cancer stage. When uCRP was combined with the urinary biomarker panel, it achieved AUCs of 0.878 (95% CI: 0.802-0.931), 0.798 (95% CI: 0.738-0.859) and 0.813 (95% CI: 0.758-0.869) in healthy vs PDAC, benign vs PDAC and healthy and benign vs PDAC sample groups, respectively. However, adding plasma CA19-9 to the urinary biomarker panel yielded a better performance, with AUCs of 0.978 (95% CI: 0.959-0.996), 0.911 (95% CI: 0.873-0.949) and 0.919 (95% CI: 0.883-0.955) in the healthy vs PDAC, benign vs PDAC and healthy and benign vs PDAC comparisons, respectively. In conclusion, we show that measuring CRP in urine is a feasible analytical method, and that uCRP could potentially be a promising biomarker in various diseases including other cancer types.

## Introduction

Pancreatic ductal adenocarcinoma (PDAC) accounts for approximately 90% of all pancreatic neoplasms (1). The majority of patients with this disease experience nonspecific symptoms and are diagnosed at an advanced stage, resulting in exceptionally poor prognosis with a five-year survival of patients with distant metastasis being only 3% (2). This dismal survival rates can be improved through early detection when the disease is still localised and amenable to surgical intervention (3, 4).

C-reactive protein (CRP) is a major acute-phase protein predominantly produced by the liver (5). It plays a vital role in the response to infection, inflammation and tissue injury where its concentration in blood increases, so it is a widely used systemic marker of severity for these conditions (5, 6). Elevated levels of blood CRP (bCRP) have been associated with an increased risk of cardiovascular disease (7) but also with increased risk of cancer in the general population (8), including non-sigmoid colon and lung cancers (9), breast, ovarian and liver cancers (10–12). The highest risk was seen in squamous cell lung cancer, where CRP levels were elevated up to 5 years before cancer diagnosis and risk of cancer rose steadily with increasing CRP levels (13).

bCRP has also been studied as a diagnostic biomarker for various types of cancer (14, 15). In PDAC, it was demonstrated that combination of bCRP and CA19-9, as well as several inflammatory cytokines can distinguish patients with PDAC from patients with chronic pancreatitis (CP) and healthy individuals (16–18). In addition, bCRP has been widely reported as a prognostic marker in a number of cancers, including PDAC (8, 19–26).

Despite CRP being one of the most commonly used biomarkers, it is typically only measured in blood samples, and there are scarce data available on measuring CRP in urine (uCRP). Chuang et al. showed that CRP is not a normal constituent of urine and is not a biomarker of local inflammation in the urinary tract (27); similarly, in two studies that measured uCRP in patients with lower urinary tract symptoms (LUTS) and children with urinary tract infections (UTI), uCRP was not found to be a specific biomarker for either condition (27, 28). In contrast, in studies by Andersson et al. (29) and Ashkenazi-Hoffnung et al. (30), urinary CRP was found to be expressed at higher levels in pediatric UTI and enabled effective differentiation of bacterial from viral urinary tract infections. Except for these reports, we could not find any additional publications on measuring CRP in urine.

We have previously described and validated a panel of urine biomarkers comprising LYVE1, REG1B and TFF1 which could detect resectable PDAC with over 90% accuracy (31, 32). We have also developed a PancRISK score for the easy interpretation of data from the panel (33). Recently, we have also demonstrated that this panel can detect PDAC up to two years prior to diagnosis (34). Here, similarly to what we have shown previously, CA19-9, a commonly used PDAC biomarker, improved the performance of the urinary panel. As CA19-9 is a blood-based biomarker, we are searching for additional urine biomarker(s) to replace it, in order to devise a wholly urine-based test for the early detection of PDAC.

It is well established that inflammation plays a crucial role in promoting tumour growth and progression (35, 36) and CRP is a widely utilised marker of ongoing inflammation. Interestingly, CRP was shown to be an independent predictor of the risk of developing type 2 diabetes (37) and it is elevated in patients with newly diagnosed diabetes mellitus (38), which are a known risk factor and an early clinical manifestation of PDAC, respectively (39). In the present study, we therefore explore the role of uCRP as a diagnostic biomarker for PDAC to determine whether it can be reliably measured in urine, and whether it can distinguish control groups from PDAC samples. Provided this is possible, we then aim to understand to what extent uCRP can improve the accuracy of our current urinary panel.

## Methods

### Clinical samples

This case-control study was performed using prospectively collected urine samples from Royal London Hospital, University College London and University of Liverpool in the UK and Cedars-Sinai in Los Angeles, USA, collected using common protocols. All the samples were collected from patients above the age of 18 who gave informed consent. The exclusion criteria were: current or prior treatment (chemotherapy, radiotherapy, surgical resection, biological therapy, and immunotherapy) for any malignancy other than basal cell carcinoma within 5 years of enrolment.

The study was approved by the Northeast - York Research Ethics Committee (18/NE/0070). In total, 534 urine samples were analysed: 93 samples forming the healthy control group were from people with no history of pancreatic conditions or malignancies at the time of collection, of these, 37 had family history of pancreatic cancer; 176 samples were collected from patients diagnosed with PDAC (123 stage I–II, 49 stage III–IV and 4 unknown), and 265 were from patients with benign hepatobiliary diseases (benign control group). This control group included patients presenting symptoms suggestive of pancreatic cancer, including, but not limited to, abdominal pain, back pain, nausea, vomiting, diarrhoea, constipation or new onset diabetes, as well as patients undergoing surgical interventions for suspected pancreatic cancer, such as cystic lesions of the pancreas. The benign sample group included: 97 samples with pancreatitis, of which 65 were chronic pancreatitis; further 54 samples were from patients with pancreatic cysts; 50 samples were from patients with biliary duct diseases such as cholecystitis and cholelithiasis; 20 samples represented various liver diseases and 44 other samples included samples collected from patients with gastritis and unspecified abdominal pain.

The demographic details of the samples are summarised in **Table 1**.

**Table 1:** Baseline characteristics of the study participants.

Characteristics	Healthy	Benign	PDAC
N	93	265	176
Gender, n (%)			
Male	36 (38.7)	134 (50.6)	97 (55.1)
Female	57 (61.3)	131 (49.4)	79 (44.9)
Age (years)			
Mean	57.6	57.9	68.6
Range	26-87	21-91	29-88
Cancer stage, n (%)			
I-II	–	–	123 (69.9)
III	–	–	20 (11.4)
IV			29 (16.5)
Unknown	–	–	4 (2.3)

### Sample preparation and analysis of CRP

Urinary CRP was measured using an ELISA kit from Immundiagnostik AG in Bensheim, Germany (Cat# K9710s; intra- and inter-assay coefficients of variation <7% and <8%, respectively; detection limit 1.015 ng/ml), according to the manufacturer’s protocol, with urine samples being diluted 1:5. The absorbance was measured with a microplate reader (FLUOstar^®^ Omega, BMG LABTECH, *Offenburg*, Germany) at 450 nm. uCRP levels were calculated from the calibration curve. Each sample was analysed in duplicate to ensure accuracy, and the average value was used for calculations. Samples below the detection limit were taken as half of the detection limit (0. 508 ng/ml). Matched blood CRP values (measured by Roche Cobas platform, Roche Diagnostics, UK) close to the urine collection date were obtained from patients’ medical records.

### Analysis of urinary biomarker panel and plasma CA19-9

Commercially available ELISA kits were used to measure the three biomarkers -TFF1, LYVE1 and REG1B- in urines as described elsewhere (31, 34). Briefly, TFF1 and LYVE1 were measured using R&D Systems (Cat# DY5237 and Cat# DY2089, respectively). Urine samples were diluted 1:10 and 1:75 for TFF1 and LYVE1, respectively. Urinary REG1B was measured with the ELISA pair set from Sino Biological Inc. (Cat# SEK11638) and urines were diluted 1:750. The substrate (TMB) and stop solution were from BioLegend (Cat# 421101 and 423001). The absorbance was determined with a microplate reader (FLUOstar^®^ Omega, BMG LABTECH, *Offenburg*, Germany) at 450 nm. Plasma CA19-9 was measured using Roche platform (Cobas 601E [ECLIA] technology) according to routine protocols. The minimum detectable level of CA19-9 was 0.3 U/ml.

### Statistical analysis

Descriptive statistics were calculated for baseline characteristics. Continuous variables were summarised as median and interquartile range (IQR) and categorical variables as frequency (percentage). Spearman correlation coefficient was used to examine the correlation of uCRP with the remaining biomarkers. Non-parametric Kruskal-Wallis test with Dunn’s correction was used to compare biomarker levels across the experimental groups.

All protein concentration data were standardised prior to the analysis. The biomarker panel was combined with age, CRP and CA19-9 in various combinations, which were analysed using receiver operating characteristics (ROC) curve to establish their ability to discriminate between PDAC and control specimens. Internal validation was performed by random splitting the whole dataset into the training and validation sets in a 1:1 ratio. Logistic regression was applied to both the training and validation sets: one group comprising of control and PDAC samples, the other comprising of benign and PDAC samples. The performance characteristics of the regression models were evaluated in the validation set and compared in terms of the area under the ROC curve (AUC). Confidence intervals (95% CIs) for AUCs were derived using 2,000 stratified bootstrap replicates.

Two-sided p-values were reported for all statistical tests; a p-value <0.05 was considered to be statistically significant. Statistical analysis was performed using IBM SPSS statistics version 28, GraphPad prism version 10 and R version 4.2.3 (https://cran.r-project.org).

## Results

**Figure 1:**
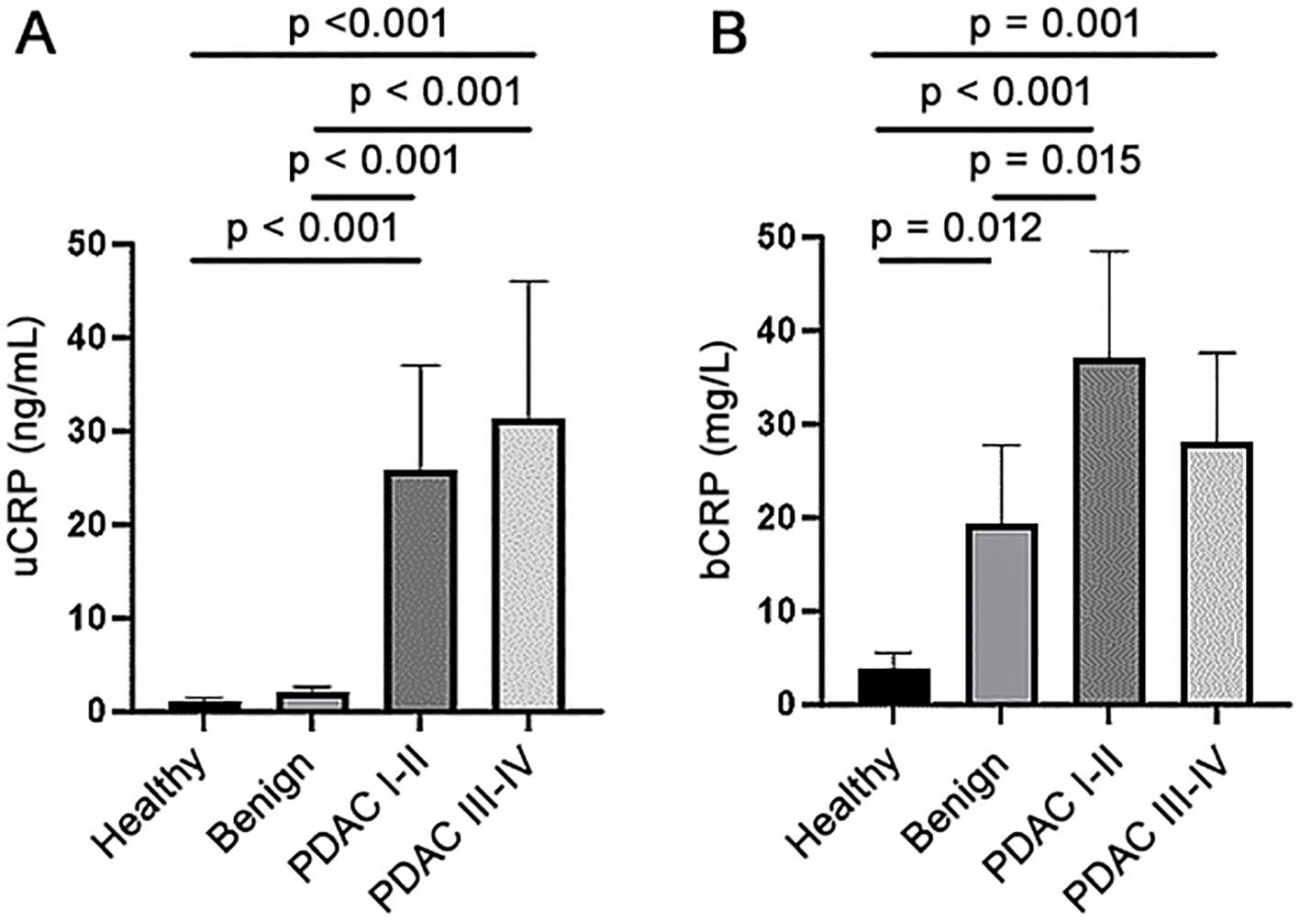
**(A)** CRP levels in urine (uCRP) and **(B)** blood (bCRP) in healthy, benign and early (stage I-II) and late stages (III-IV) PDAC samples.

**Table 1** presents the baseline characteristics of the study participants. Of the healthy and benign controls, only two (2.2%) and 21 participants (7.9%), respectively had uCRP levels above the detection limit, while this was the case in 73 PDAC patients (41.5%). When the cut-off value of 6ng/mL was used, the specificity (SP) of CRP was 99% and 94% (for healthy controls and healthy and benign controls combined, respectively) and sensitivity (SN) was 30%. The uCRP level was significantly higher (p < 0.001) in participants with PDAC than in both control groups (**Figure 1A**, **Table 2**). Values for bCRP were available only for a subset of samples, but the performance was very similar to uCRP (**Figure 1B**, **Table 2**). Of note, no significant difference was found in uCRP levels between males and females, and age did not show a significant correlation with uCRP in either the PDAC or control groups. As shown previously, CA19-9 and all three urinary biomarkers were significantly higher in PDAC compared to the control groups (**Table 2**). A significant but weak correlation (r = 0.353, p < 0.001) was found between uCRP and bCRP levels; uCRP also weakly correlated with CA19-9, LYVE1 and TFF1 (r = 0.302, r = 0.341 and r = 0.358, respectively, p < 0.001) (**Supplementary Figure S1**).

**Table 2:** Biomarker concentrations across different groups. Values are presented as median and IQR.

Markers	Healthy	Benign	PDAC	P-value ^		
	Median (IQR)	Median (IQR)	Median (IQR)	H vs B	H vs PDAC	B vs PDAC
uCRP (ng/mL)	0.508 (0.508-0.508)	0.508 (0.508-0.508)	0.508 (0.508-7.34)	0.717	<0.001	< 0.001
bCRP (mg/L)*	1.55 (0.78-3.13)	3.5 (1.9-13.5)	12.5 (3.5-45)	0.012	<0.001	0.003
CA19-9 (kU/L)	5 (1.2-8)	13 (7-25)	217 (41-981)	< 0.001	<0.001	<0.001
REG1B (ng/mL)	9.88 (4.95-31.52)	19.86 (5.85-62.13)	105.84 (25.28-500)	0.053	<0.001	<0.001
LYVE1(ng/mL)	4.44 (0.4-17.04)	12.39 (3.92-28.77)	36.4 (16.23-92.6)	< 0.01	< 0.001	< 0.001
TFF1 (ng/mL)	0.23 (0.04-1.08)	0.83 (0.25-1.77)	2.7 (1.39-5.1)	< 0.01	< 0.001	< 0.001

*Values available for n=24 Healthy, n= 111 Benign and n=33 PDAC. ^P-values are obtained from Kruskal-Wallis Test with Dunn’s correction. uCRP, CRP in urine; bCRP, CRP in blood.

**Figure 2:**
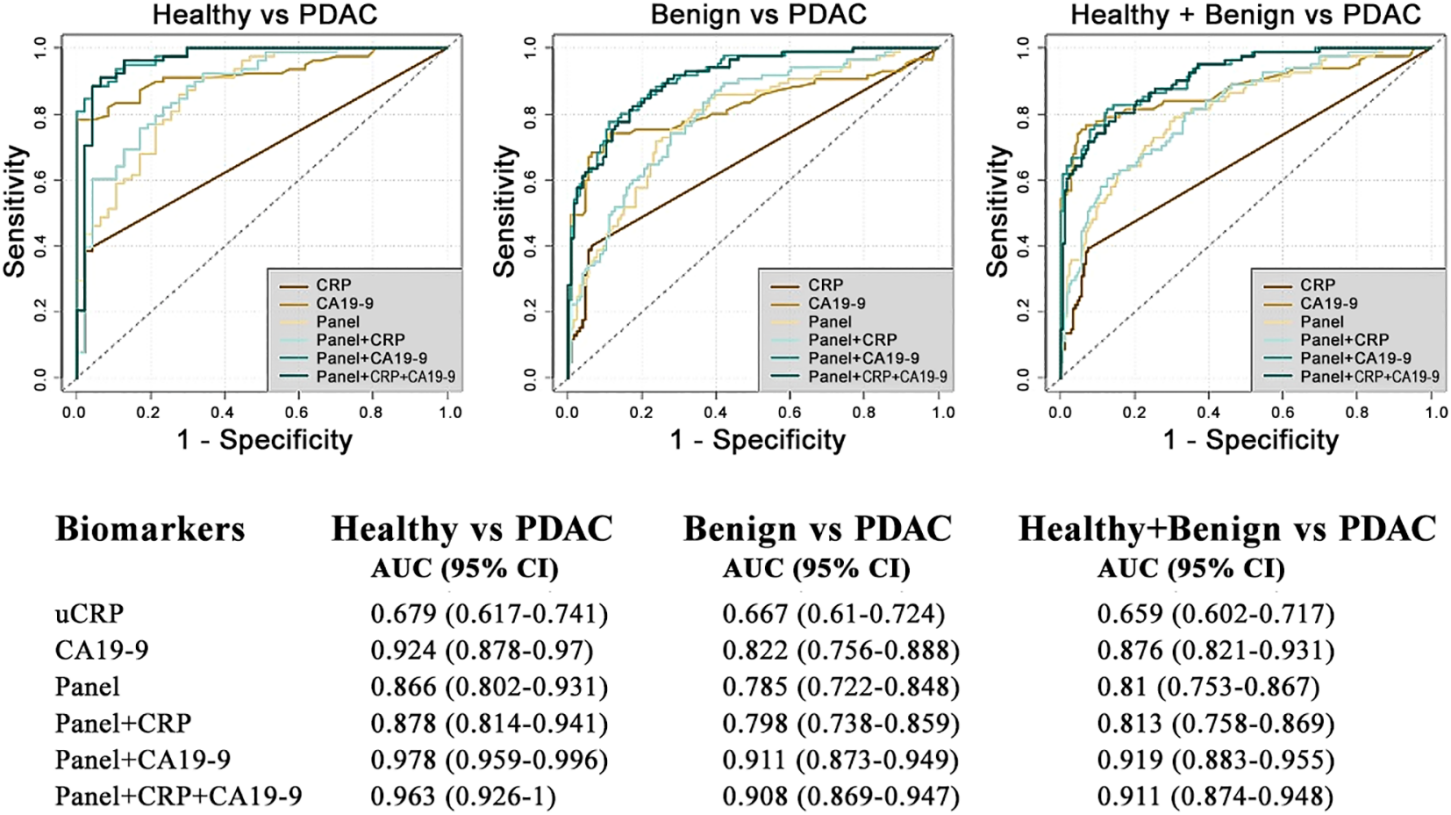
Diagnostic performance of biomarkers discriminating patients with PDAC from benign and healthy controls.

The performance of uCRP alone and in combination with our urinary panel (REG1B, LYVE1 and TFF1 + age), and CA19-9 were assessed using the receiver operator characteristic (ROC) curve analysis (**Figure 2**). The uCRP alone resulted in AUCs of 0.679 (95% CI: 0.617-0.741) for healthy vs PDAC, 0.667 (95% CI: 0.610-0.724) for benign vs PDAC and 0.659 (95% CI: 0.602-0.717) for healthy + benign vs PDAC. When uCRP was combined with the urinary biomarker panel, it enhanced the performance with AUCs to 0.878 (95% CI: 0.802-0.931), 0.798 (95% CI: 0.738-0.859) and 0.813 (95% CI: 0.758-0.869) in healthy vs PDAC, benign vs PDAC and healthy + benign vs PDAC sample groups, respectively. However, the combination of plasma CA19-9 with the urinary panel yielded a superior performance, with AUCs of 0.978 (95% CI: 0.959-0.996), 0.911 (95% CI: 0.873-0.949) and 0.919 (95% CI: 0.883-0.955) in the healthy vs PDAC, benign vs PDAC and healthy + benign vs PDAC sample groups, respectively. Further addition of uCRP to plasma CA19-9 and the biomarker panel did not result in any further improvement (AUCs of 0.963 (95% CI: 0.926-0.1.00), 0.908 (95% CI: 0.869-0.947), 0.911 (95% CI: 0.874-0.948) for healthy vs PDAC, benign vs PDAC and healthy + benign vs PDAC sample groups, respectively).

## Discussion

Since its discovery in 1930 as a protein that reacts with C-polysaccharide on Streptococcus pneumoniae (40), elevated levels of CRP have been associated with both acute and chronic systemic inflammation. As inflammatory milieu can both favour cancer development and progression and can be induced by tissue injury during cancer growth and spread. Increased CRP levels have been associated with an increased risk of cancer as well as being a harbinger of poor prognosis for this disease (8, 9, 41).

The detailed mechanistic roles of CRP in cancer are still being unravelled. CRP exists in two forms: the circulating pentameric CRP (pCRP) and the monomeric isoform (mCRP). pCRP is highly soluble and rapidly dissociates into mCRP, which is less soluble and self-aggregates in tissues (14). mCRP binds to membrane lipids triggering complex intracellular signalling via phospholipase C, MAPK, ERK, Akt, STAT and NFkB, which are important effector pathways strongly associated with the intrinsic malignant characteristics of cancer cells (42). In addition, mCRP can also modulate inflammatory responses through stimulating platelets, leukocytes and complement system as a response to tumour growth (14).

The production of CRP in hepatocytes is regulated by cytokines, particularly by IL-6, which is secreted by macrophages, endothelial and other cells activated after local tissue injury, leading to an increase in pCRP concentrations in the bloodstream that is being measured (43).

In PDAC, combining bCRP with other biomarkers was shown to be able to differentiate PDAC from benign and control groups, thus aiding the early detection of PDAC: Zhang et al. showed that a panel of CRP with CA19-9, albumin and IL-8 had high diagnostic value for distinguishing PDAC and healthy controls with 96% SN at 90% SP in early stage disease, while CRP combined with CO_2_, CA19-9 and IL-6 discriminated between PDAC and benign cases with 75% SN at 90% SP (18). Furthermore, Lanki et al., showed that combining bCRP with CA19-9 and inflammatory cytokines CTACK, GRO-a, b-NGF may aid in distinguishing PDAC from CP, but the combination was not superior to CA19-9 alone (17). Ferri et al., tested CA19-9, CRP, IGF-1, CEA and albumin alone and in various combinations (16) and when a CRP cut-off value of 2.3 mg/L was used, SN of 76.6% and a SP of 55% for differentiating between PDAC and CP was established. Notably, significantly higher levels of CRP were seen in jaundiced PDAC patients, suggesting its potential role in distinguishing jaundiced PDAC from CP patients (16). Higher bCRP concentration in PDAC than in CP was also reported by Mroczko et al. (44). All of these studies measured CRP in blood, and with the exception of very few studies (27, 30, 45), hardly any attempt was made to measure CRP in urine. To the best of our knowledge, we could not find any reports on the use of uCRP as a potential diagnostic or prognostic marker for any cancer, including PDAC.

Our long-standing goal is to develop a non-invasive test for early detection of PDAC using urine samples. We chose urine because it is a less complex matrix than blood, with a lower dynamic range, which can be easily, repeatedly and completely non-invasively self-sampled (46, 47).

Limited information is available on CRP kinetics in humans. bCRP is pentameric, and its conversion to mCRP is rapid, with a lag of approximately 6-12 hours before the increase in CRP is seen in blood; clearance of bCRP is largely through renal filtration, as shown by monitoring of excretion of ^125^I-CRP in a study by Vigushin et al., where a similar rate of excretion was seen in both normal and diseased volunteers (48). While it is not yet clear which form of CRP is excreted into human urine, it has been shown that the binding of calcium and the chelation of proteolysis-resistant pCRP causes considerable protein compaction leading to a significant reduction of its Stoke’s radius (49), which could enable the filtration of this otherwise large (approximately 120kDa) protein through the glomerular barrier. Alternatively, as shown in mouse models, both pCRP and mCRP can be excreted in urine (50), so it is possible that the otherwise proteolysis-sensitive and poorly soluble mCRP is contained within microvesicles that are shed from activated cells during an inflammatory response.

Regardless of this, we found a weak but positive correlation of uCRP with bCRP, which was also shown in one of the rare studies that explored CRP in both urine and blood samples in patients with pneumonia (45). This suggests that completely non-invasive measuring of CRP using urine samples is a feasible option.

This study presents the first evaluation of uCRP in cancer, where the main aim was to establish whether uCRP can be used as a biomarker for early detection of PDAC. We show that uCRP exhibits a very high SP but a relatively low SN in distinguishing between healthy and benign controls from PDAC samples. Combining uCRP with our urine biomarker panel resulted in a slight increase of the AUC, particularly in healthy vs PDAC comparison, however, the addition of plasma CA19-9 to the panel led to a superior performance, which was not further improved by adding uCRP.

The main strength of our study is that we analysed uCRP levels along with our urinary panel biomarkers in control, benign, and PDAC samples from four different centres, including a large number (70%) of early stage PDACs. The significant limitation of our study was that matched bCRP values were obtained from clinical notes, rather than being re-measured.

In conclusion, we have demonstrated that measuring CRP in urine is a straightforward and feasible method. Consequently, this suggest that uCRP could potentially be a promising biomarker for detecting other diseases, including other cancer types, which warrants further confirmation in future studies.

## Data Availability

The raw data supporting the conclusions of this article will be made available by the authors, without undue reservation.
