# Supplementary material for: Evaluation of urinary C-reactive protein as an early detection biomarker for pancreatic ductal adenocarcinoma
Source: Front Oncol. 2024 Sep 6;14:1450326. doi: 10.3389/fonc.2024.1450326 (PMC11412792; doi:10.3389/fonc.2024.1450326)
Supplement: Supplementary file 1 [file DataSheet1.docx]

**Evaluation of urinary C-reactive protein as an early detection biomarker for pancreatic ductal adenocarcinoma**

**Short title: urinary C-reactive protein and pancreatic ductal adenocarcinoma**

Nurshad Ali^1^, Silvana Debernardi^1^, Evelyn Kurotova^1^, Jian Tajbakhsh^2,3^, Nirdesh K Gupta^2^, Stephen J. Pandol^4^, Patrick Wilson^5^, Stephen P. Pereira^6^, Bill Greenhalf^7^, Oleg Blyuss^8,9^, Tatjana Crnogorac-Jurcevic^1^

**Corresponding authors**

Nurshad Ali ([nurshad.ali@qmul.ac.uk](mailto:nurshad.ali@qmul.ac.uk))

Tatjana Crnogorac-Jurcevic ([t.c.jurcevic@qmul.ac.uk](mailto:t.c.jurcevic@qmul.ac.uk))

*r* = 0.302

*p* < 0.001

*r* = 0.353

*p* < 0.001


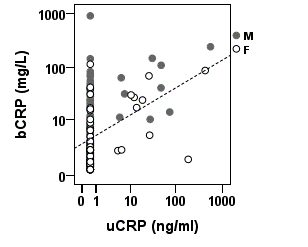

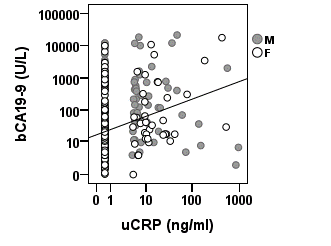

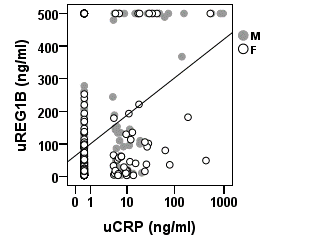

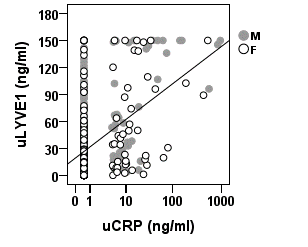

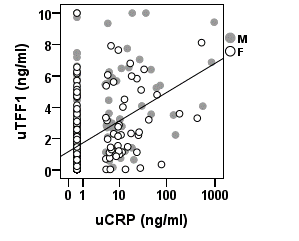


**A**

**B**

**D**

**C**

**E**

**B**

*r* = 0.341

*p* < 0.001

*r* = 0.358

*p* < 0.001

*r* = 0.271

*p* < 0.001

**Figure S1** Correlation of uCRP with other biomarkers: CRP in blood (n=168) (A), CA19-9 in blood (B), REG1B in urine (C), LYVE1 in urine (D) and TFF1 in urine (E). P-values are obtained from the Spearman correlation coefficient.
